# A Local Weighted Nearest Neighbor Algorithm and a Weighted and Constrained Least-Squared Method for Mixed Odor Analysis by Electronic Nose Systems

**DOI:** 10.3390/s101110467

**Published:** 2010-11-18

**Authors:** Kea-Tiong Tang, Yi-Shan Lin, Jyuo-Min Shyu

**Affiliations:** 1 Department of Electrical Engineering, National Tsing Hua University / No. 101, Sec. 2, Kuang-Fu Road, Hsinchu, 30013, Taiwan; 2 Department of Computer Science, National Tsing Hua University / No. 101, Sec. 2, Kuang-Fu Road, Hsinchu, 30013, Taiwan; E-Mails: sandy54013@gmail.com (Y.-S.L.); shyu@cs.nthu.edu.tw (J.-M.S.)

**Keywords:** electronic nose, mixed odor analysis, weighted nearest neighbor, weighted and constrained least-squared method

## Abstract

A great deal of work has been done to develop techniques for odor analysis by electronic nose systems. These analyses mostly focus on identifying a particular odor by comparing with a known odor dataset. However, in many situations, it would be more practical if each individual odorant could be determined directly. This paper proposes two methods for such odor components analysis for electronic nose systems. First, a K-nearest neighbor (KNN)-based local weighted nearest neighbor (LWNN) algorithm is proposed to determine the components of an odor. According to the component analysis, the odor training data is firstly categorized into several groups, each of which is represented by its centroid. The examined odor is then classified as the class of the nearest centroid. The distance between the examined odor and the centroid is calculated based on a weighting scheme, which captures the local structure of each predefined group. To further determine the concentration of each component, odor models are built by regressions. Then, a weighted and constrained least-squares (WCLS) method is proposed to estimate the component concentrations. Experiments were carried out to assess the effectiveness of the proposed methods. The LWNN algorithm is able to classify mixed odors with different mixing ratios, while the WCLS method can provide good estimates on component concentrations.

## 1. Introduction

An electronic nose is a biomimetic olfactory system developed based on chemical sensor principles, electronic system design and data analysis techniques. In the biological olfactory system, there are about 350 different odorant receptors in humans and about 1,000 in mice. Different odors are recognized by different combinations of odorant receptors [1,2]. Learning from this mechanism, an array of different chemical sensors is used in the design of an electronic nose. An odor can be identified by classifying its response pattern generated by the sensor array in the electronic nose [3–5].

The state-of-the-art techniques for sensor array data analysis and the applicability of each technique have been discussed by Jurs [6]. One type of data analysis methods is classification, which aims to group an object into one of the predefined class. K-Nearest Neighbor classifier (KNN) is one of the widely applied classification method that classifies an item according to the majority voting of the K nearest items. Instead of setting a global value for K, Locally Adaptive Nearest Neighbor (Local KNN) computes a locally varying K value for each query point by using the information from the neighbors of the query point [7]. On the other hand, since features may not be equally effective for classification, Discriminant Adaptive Nearest Neighbor (DANN) uses a locally weighted distance measurement scheme to compute the distance between two points [8]. The accuracy of KNN and its two variants, Local KNN and DANN, were examined by Bicego [9]. These three KNN-based methods were comparable on the examined data sets regardless of the computational cost.

The methods of dimensionality reduction, such as Principal Component Analysis (PCA) and Linear Discrimination Analysis (LDA), seek to reduce the data size required for classification. PCA is an unsupervised method, which finds a set of orthogonal projection directions that capture the largest amount of variation in data without using the class information of the data. On the other hand, LDA makes use of the class labels to find a lower-dimensional vector space for best class separation. For example, a 100% classification rate was achieved by LDA for classification of different tomato maturity states and different qualities of green tea samples [10,11]. The study in [12] indicates that PCA could yield superior classification results when a small training set is used. However, traditional classification methods would require significant computational cost if the sensor number is large.

Regression analysis is a statistical data analysis approach which seeks a continuous fitting function of independent variables to model the dependent variables. The Least-Squares method can be used to find such fitting function by minimizing the sum of squared differences between each of the known data point and the fitting function. The NASA’s Jet Propulsion Laboratory (JPL) used a set of self-developed polymer composite sensors to quantify single and mixed contaminants [13,14]. A second order polynomial regression based on the assumption of additive linearity was used to model the relationship between the gas concentration vector and the sensor responses. Carmel *et al*. [15] took the same assumption and further considered the relative influence of each component on the total mixture response. The modified model provided a promising result when more than two components were present in the examined mixture.

Although the classification methods represent a promising technology for analyzing electronic nose data, its applications are mainly focused on discrimination between different odors. Moreover, odors containing the same components but with different mixing ratios are generally perceived as different smells. For this reason, a traditional classification method will not be applicable for differentiating the smells. A more practical solution is to partition the odor space into subspaces and classify an odor into one of the subspaces. This paper adopts a supervised strategy to categorize the mixed odor dataset into several groups according to the components. The nearest neighbor method is then used to classify the response pattern into one of the predefined groups. A weighting scheme is proposed to re-scale the distance between two data points and thus the classification accuracy could be improved. Another solution for analysis of odor mixture is to directly determine the concentration of each component present in the examined mixture by analyzing the response pattern. Regression methods are applied in this paper to build odor models. The component concentrations are estimated by solving a weighted and constrained least-squares problem, in which each of the squared error term is weighted to reflect the reliability of each estimated sensor response.

The rest of this paper is organized as follows: Firstly, the proposed methods for analyzing mixed odors will be described in Section 2. Then, the data collection methods and experimental results will be provided to evaluate and support the proposed methods in Section 3. Finally, Section 4 will conclude the contribution of this work.

## 2. The Proposed Analysis Methods

Traditionally, an electronic nose is not designed to analyze mixed odors but merely to differentiate between different smells. This paper proposes to determine the components that are most significant in a mixture by analyzing the sensor response pattern of the odor mixture. This work is based on the following two assumptions [13–15]:

♦ *Homogeneity*: The sensor response to an odor is proportional to the odor concentration.
♦ *Linear Additive*: The sensor response to a mixture is equal to the linear summation of the sensor response to each of its components.

**Figure 1.**
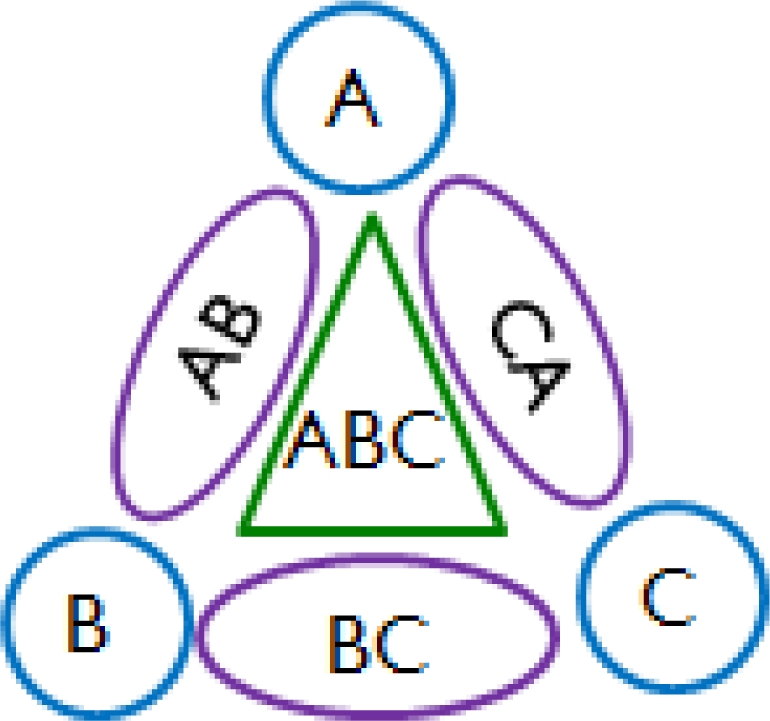
A schematic plot of the normalized data set of mixed odors consists of three odor components: A, B and C. The data points are partitioned into seven component sets according to the contained odor components: A, B, C, AB, BC, CA, and ABC.

Based on the assumption of homogeneity, the normalized mixed odor dataset could be categorized according to the contained components without considering the concentration of each component. For example, the categorization results for odors of three components would be like the one shown in Figure 1. The response pattern of the examined odor would then be classified to one of the predefined classes by using a classification method. However, the sensors may not provide enough useful information sufficient enough to classify an odor. A method of dimensionality reduction, such as PCA and LDA, can then be applied to select the significant features to achieve a better result of data partition. However, both PCA and LDA require solving a complex matrix eigenvalue problem in order to find the projection directions. In this paper, a simple local weighting scheme is proposed to properly weight each feature for a class.

### 2.1. Locally Weighted Nearest Neighbor (LWNN)

Assume that there are *N* predefined classes. In the nearest neighbor classification method, which is actually a KNN method with *K* = 1, a predefined class *Class_i_* is represented by its centroid *o⃑_i_*, where 1 ≤ *i* ≤ *N* and *m* is the number of sensors. Here, we define the class centroid as the mean point of class:

(1) $$o_{i,j}=\frac{1}{\left|{\mathrm{Class}}_{i}\right|}\sum_{\vec{p}\in {\mathrm{Class}}_{i}}p_{j}$$

where *o_i,j_* is the *j*th component of *o⇀_i_*. A testing point is defined as the class of the nearest centroid. In the proposed weighting scheme, instead of directly computing the Euclidean distance between the testing point *x⇀* and the examined centroid *o⇀_i_*, an independent weighting vector *ω⇀_i_* is associated to each class to re-scale the Euclidean distance, *i.e.*:

(2) $$d(\vec{x},{\vec{o}}_{i})=\sqrt{\sum_{j=1}^{m}\omega_{i,j}\cdot {\left(x_{j}-o_{i,j}\right)}^{2}}$$

where *m* is the number of sensors. For each class the weighting vectors are determined by minimizing the num of squared weighted distance from each training data point to the centroid of its belonged class, that is:

(3) $$\min_{\omega_{1},\omega_{2},\ldots ,\omega_{N}}\sum_{i=1}^{N}\sum_{\vec{p}\in {\mathrm{Class}}_{i}}d^{2}(\vec{p},{\vec{o}}_{i})$$

subject to $\prod_{j=1}^{m}\omega_{i,j}=1$, for 1 ≤ *i* ≤ *N*. The optimization problem in Equation (3) can be solved by *Lagrangian Multipliers*. The optimal weighting vector associated to each class is computed as:

(4) $$\omega_{i,j}=\frac{\lambda_{i}}{\sum_{\vec{p}\in {\mathrm{Class}}_{i}}{\left(p_{j}-o_{i,j}\right)}^{2}}$$

where:

$$\lambda_{i}={\left(\sum_{j=1}^{m}\sum_{\vec{p}\in {\mathrm{Class}}_{i}}{\left(p_{j}-o_{i,j}\right)}^{2}\right)}^{\frac{1}{m}}$$

Note that Equation (4) indicates that is the points belonging to the same class exhibit a string correlation in *j*th feature, a large weight would be assigned to this feature for the class. Aside from these observations, the optimal weighting vectors can be computed without too much effort since the computation of each weighting term is expressed in a closed-form.

As aforementioned, the proposed *Locally Weighted Nearest Neighbor* algorithm (LWNN) uses the weighting scheme to re-scale the Euclidean distance between two data points when finding the nearest neighbor. Unlike the original KNN algorithm, the proposed LWNN algorithm has a training stage, which computes both the centroid and the associated independent weighting vector of each predefined class (Table 1). Then, as shown in Table 2, LWNN classifies a testing data point as the class of the centroid that has the minimum weighted distance to the examined point. Note that it is unnecessary to take any additional step to determine the best *K* value so as to increase the classification accuracy. In practice, as it will be shown later in Section 3, the experimental results of determining the component set demonstrates that the accuracy of the proposed LWNN classifier is comparable to that of those commonly used KNN-based methodologies.

### 2.2. Odor Concentration Estimation by Weighted Least-Squares Method

Although the proposed LWNN method can be used to efficiently determine the set of components present in an odor mixture, the concentration of each component is still unknown. Nevertheless, a regression method could be used to estimate the component concentration. According to the assumption of homogeneity, the sensor generated by the *i*th sensor to the *j*th odor component at a concentration c_j_ can be formulized as:

(5) $$r_{i}(c_{j})=\alpha_{i,j}\cdot c_{j}$$

Based on the linear additive assumption, the response of the *i*th sensor when exposed to an odor mixture consists of *n* components with concentrations *c*_1_, *c*_2_,..., *c_n_*, respectively, can be formulized as:

(6) $$r_{i}(c_{1},c_{2},...,c_{n})=\beta_{i,\mathrm{offest}}+\sum_{j=1}^{n}\beta_{i,j}\cdot r_{i}(c_{j})$$

Note that the response of each component is weighted with a weighting term β_i,j_ and an offset term β_i,offest_ is introduced in Equation (6) to get a better fit for the sensor responses. According to [15], this weighting scheme on the response of each component can be seen as a reflection of the relative influence of each component on the total response.

The parameters in Equation (6) could be obtained by applying a method for linear least-squares problems. Then, the concentration of each mixture component can be estimated by solving the following least-squares formulation:

(7) $$\min_{c_{1},c_{2},...c_{n}}\frac{1}{2}\sum_{i=1}^{m}{\left(r_{i}(c_{1},c_{2},...,c_{n})-t_{i}\right)}^{2}$$

subject to:

$$c_{1},c_{2},...,c_{n}\geq 0$$

where *n* is the number of the components, *m* is the number of sensors, and *t_i_* is the *i*th sensor response of the examined odor mixture. The nonnegative constraints are introduced in Equation (7) in order to get a feasible solution. Moreover, to reflect the effectiveness of the estimated sensor response, a weighting scheme on each sensor response is proposed to properly weight each squared error term in Equation (7), and thus the following formulation is to be solved:

(8) $$\min_{c_{1},c_{2},...,c_{n}}\frac{1}{2}\sum_{i=1}^{m}\omega_{i}\cdot {\left(r_{i}(c_{1},c_{2},...,c_{n})-t_{i}\right)}^{2}$$

subject to:

$$c_{1},c_{2},...,c_{n}\geq 0$$

In order to get a close form expression for each of the weighting terms, the product of the weighting terms is set to one:

$$\prod_{i=1}^{m}\omega_{i}=1$$

According to Equation (4), the weighting term of the *i*th sensor is defined as:

(9) $$\omega_{i}=\frac{\lambda }{\sum_{k=1}^{\left|T\right|}{\left(r_{i}\left(c_{1}{}^{\left(k\right)},c_{2}{}^{\left(k\right)},\ldots ,c_{n}{}^{\left(k\right)}\right)-t_{i}{}^{\left(k\right)}\right)}^{2}}$$

and:

$$\lambda ={\left(\prod_{i=1}^{m}\sum_{k=1}^{\left|T\right|}{\left(r_{i}\left(c_{1}{}^{\left(k\right)},c_{2}{}^{\left(k\right)},\ldots ,c_{n}{}^{\left(k\right)}\right)-t_{i}{}^{\left(k\right)}\right)}^{2}\right)}^{\frac{1}{m}}$$

where |*T*| is the number of the training data, *m* is the number of sensors, and *t_i_*^(*k*)^ is the *i*th observed sensor response of the *k*th training data. Equation (9) indicates that if the predicated sensor response is close to the observed sensor response, a higher weight will be assigned to that response.

The proposed methodology that uses a weighted and constrained least-squares method (WCLS) to estimate the component concentrations of a mixed odor is presented in Table 3 and Table 4. In the training stage, a set of odor models for both pure and mixed odors are built by using the least-squares method. Moreover, a set f weighting terms are computed and then used in the testing stage to estimate the concentration of each component present in an odor mixture.

Although mixing of odors can yield linear additive trend, it is not necessarily common. The effect of mixing can often lead to (1) masking or dominance by a stronger component [16], (2) hypoadditivity (lower than the sum or average) [17,18], and (3) synergistic effects [19,20].

## 3. Experimental Results and Discussion

**Figure 2.**
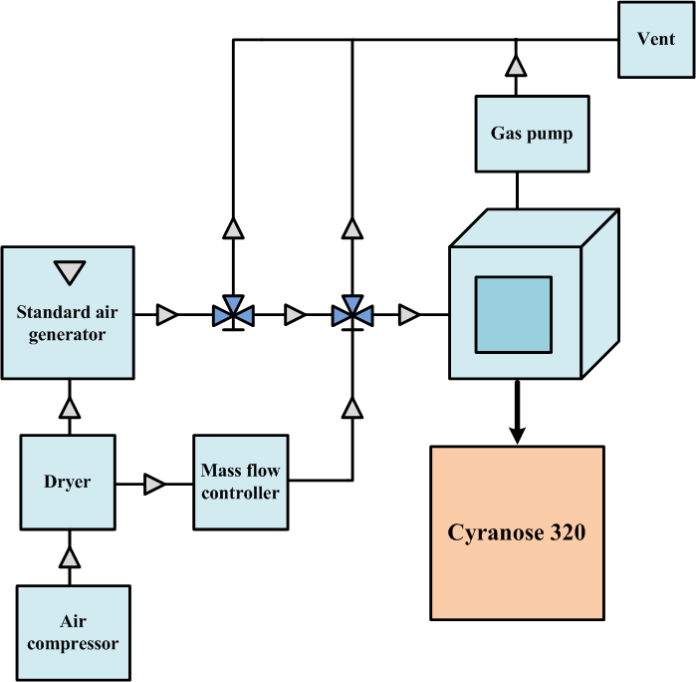
A schematic plot of the experimental setup for data collection.

Figure 2 shows the experimental setup used to collect the volatile organic compound (VOC) for analysis. The target gas for the test was produced by a standard air generator (AID360). The solvent of the testing gas sat inside the diffusion tube of the standard air generator under room temperature. A constant heater was used to increase the temperature in the tube to cause the organic solvent to evaporate. By the time the whole system reached steady temperature and flow rate for the whole system, a testing gas with stable concentration was achieved. Diffusion rate can be theoretically controlled by the temperature setting, and air concentration can be accurately calculated by measuring the weight loss of the organic solvent. The testing gas was carried out by steady air coming from the air compressor. The gas flow rate was controlled by the mass flow controller (MFC). The testing air was then infused into the glass chamber, which connects to a commercial Cyranose 320 electronic nose, which consists of 32 carbon black composite sensors. After completing the experiment, the testing air was pumped out to a Fourier transform infrared spectrophotometer (FTIR) with built-in database for cross-validation, and dry air was again used to purge the chamber. A collection of 133 mixed odor data collected by Cyranose 320 was uploaded to a personal computer after the experiment for further analysis. Three highly volatile solvents: methanol, ethanol and acetone, were mixed with different mixing ratios by using multiple air generators and mass flow controllers. The collected data are randomly divided into two sets, called the training set and the testing set, each of which contains 67 and 66 odor data, respectively. Since there are eight different types of sensors in the Cyranose 320, eight response features are derived by averaging the responses generated by four sensors of the same type in order to get a more stable sensor response. That is to say, an odor is represented by the odor pattern formed from eight averaged sensor responses.

**Figure 3.**
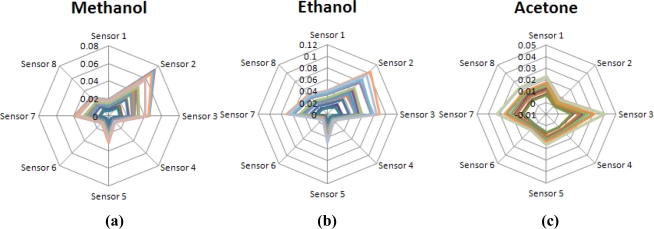
The normalized odor patterns of three vaporized solvents with different concentrations: (a) methanol, (b) ethanol and (c) acetone.

Figure 3 shows the normalized odor patterns of the examined components: methanol, ethanol and acetone, with different concentrations. As shown, the normalized odor pattern of acetone is quite different to those of the others. However, both methanol and ethanol have almost the same normalized odor pattern because these two compounds are very similar in chemical structures and intermolecular forces.

**Figure 4.**
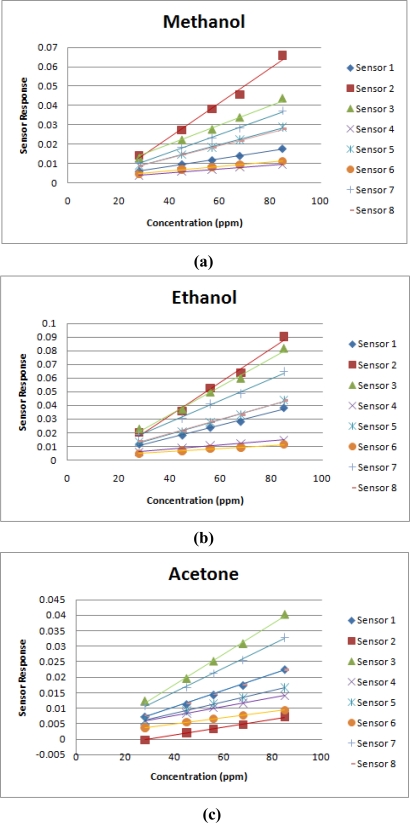
The response of each sensor over three vaporized solvents: **(a)** methanol, **(b)** ethanol and **(c)** acetone, under different concentrations.

Figure 4 shows the relationship between the sensor responses and the concentrations of the examined components and the regression line. As expected, the sensor responses are proportional to the odor concentrations. Consequently, both of the proposed LWNN method (Section 2.1) and the odor model of single compound (Section 2.2) are supported by the confirmation of the homogeneity assumption.

### 3.1. Odor Component Determination Results

This section presents the performance of the KNN-based methodologies, which are listed below:

1. KNN: KNN using the default Euclidean distance metric.
2. PCA+KNN: KNN over the reduced space generated by Principal Component Analysis (PCA).
3. LDA+KNN: KNN over the reduced space generated by Linear Discrimination Analysis (LDA).
4. WNN: The proposed Locally Weighted Nearest Neighbor method.

For each method, except for LWNN, in which the K value is fixed to one, the value of K varies from one to five. The performances of the four KNN-based methods were evaluated by using the collected odor data. The training dataset were partitioned into seven component sets according to the components:

♦ M: methanol.
♦ E: ethanol.
♦ A: acetone.
♦ ME: mixture of methanol and ethanol.
♦ EA: mixture of ethanol and acetone.
♦ AM: mixture of acetone and methanol.
♦ MEA: mixture of methanol, ethanol and acetone.

The results are summarized in Table 5. For each method, the K value that provided the best performance on the testing set is marked. As shown, the LDA + KNN strategy outperforms the other methods over the collected odor data set; while PCA has the worst performance.

**Figure 5.**
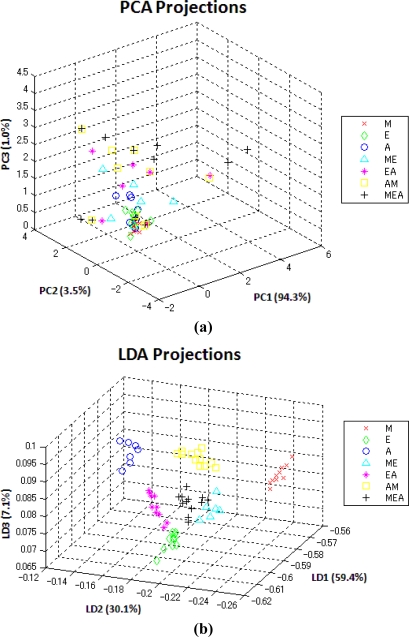
The projections of **(a)** PCA and **(b)** LDA over the testing set.

The reason is that PCA seeks to separate all the data points as widely as possible. However, the local correlation structure of each component set may be distorted. As shown in Figure 5, the method of PCA widely distributes all the data points while they are mixed together. In contrast, LDA can discriminate between different classes and keep the data points of the same class as compact as possible. Note that the projections of LDA over the testing dataset in Figure 5 match up the seven partitions in Figure 1.

Although the method of KNN applied with LDA outperforms the proposed LWNN method; LWNN is the most efficient way among the examined KNN-based methods since there is no additional computation to determine the best K value. Moreover, LWNN does not require solving any costly eigenvalue problem, which is necessary for both PCA and LDA. Nevertheless, the proposed LWNN method yields an acceptable accuracy to classify and identify the component set.

### 3.2. Estimation Results for Mixed Odors

This section reports the performance of the proposed methodology that uses a weighted and constrained least-squares method to estimate the concentration of each component present in an odor mixture. The randomly assigned training dataset are used to build odor models:

1. P_M_: the odor model for methanol.
2. P_E_: the odor model for ethanol.
3. P_A_: the odor model for acetone.
4. M: the odor model for mixtures of methanol, ethanol and acetone.

Two methodologies for estimating component concentrations are tested and compared in this section:

1. CLS: the constrained least-squares method.
2. WCLS: the proposed weighted and constrained least-squares method.

The metric of *Root Mean Squared Error* (RMSE) is adopted to evaluate the error between the estimated component concentration (*c*_*E*_1__, *c*_*E*_2__, ... *c_E_n__*) and the real component concentrations (*c*_*R*_1__, *c*_*R*_2__, ... *c_R_n__*), *i.e.*,

$$\mathrm{RMSE}=\sqrt{\frac{\sum_{i=1}^{n}{\left(c_{E_{i}}-c_{R_{i}}\right)}^{2}}{n}}$$

where *n* is the number of components.

**Figure 6.**
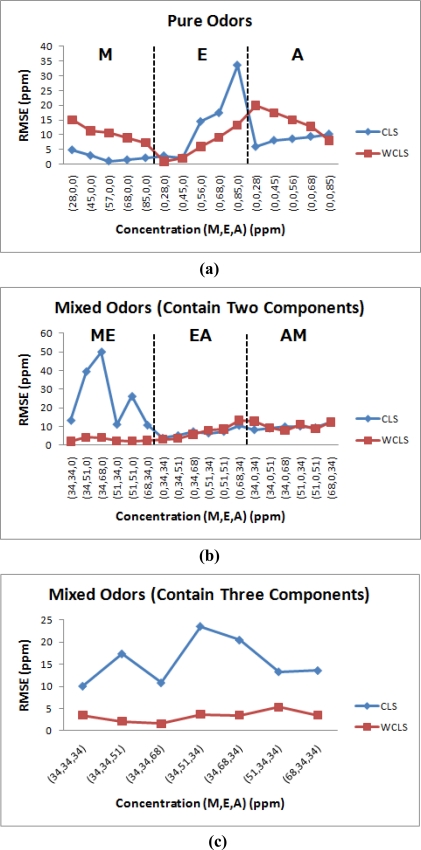
The estimated errors of the CLS method and the proposed WCLS over the testing dataset for **(a)** pure odors, **(b)** mixed odors (two components), and **(c)** mixed odors (three components).

**Figure 7.**
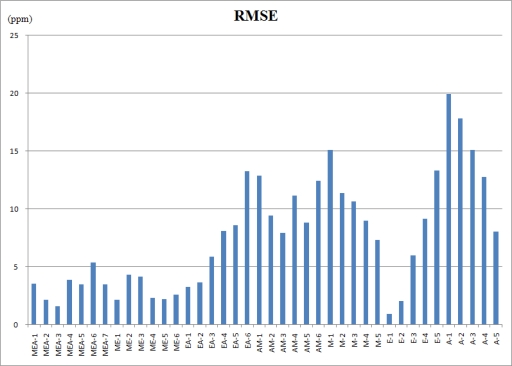
The root mean squared error of all the estimated results in Tables 6–8.

Figure 6 shows the estimated errors of the regular constrained least-squares method (CLS) and the proposed weighted and constrained least-squares method (WCLS) over the testing odor dataset. The error presented is the averaged error for each concentration combination. As shown, the proposed WCLS methodology generally produces much better estimates compared to the other method: the error curves of WCLS are almost always lower than those of CLS especially for mixed odors. As presented in Table 6, the maximum error for estimate of mixtures containing all the three components is no more than 6 ppm. However, when the number of components decreases, the estimate result becomes worse (Table 7 and Table 8). Figure 7 shows the root mean squared error (RMSE) of all the estimated results in tables 6–8. The reason is attributed to the proposed weighting scheme which assigns a larger weight to the responses of sensors 1, 3 and 8, where the responses of methanol and acetone are similar to those of ethanol. Consequently, for the component set of A, the proposed WCLS methodology could not differentiate between ethanol and acetone when the concentration of acetone is low. Moreover, since the patterns of both ethanol and methanol are quite similar as we have seen in Figure 3, the proposed methodology would be confused. Therefore, for the four component sets: M, E, EA and AM the estimate would report a high concentration of methanol accompanied with a low concentration of ethanol, and vice versa. Nevertheless, when both methanol and ethanol are present in the examined odor, the proposed WCLS could provide a good concentration estimate.

## 4. Conclusion

This study aimed to determine the mixture components and estimate the concentration of each of the contained component, assuming homogeneity and linear additive. A KNN-based method, LWNN, is proposed to determine the components present in a mixed odor by classifying its sensor responses to the closest previously partitioned component sets. Furthermore, a local weighting scheme, which associates each component set with an independent weighting vector, is proposed to re-scale the distance between a testing data point and the centroid of a component set. For each component set, a higher weight is assigned to the sensor response when the sensor yields a very consistent response to that class.

To further estimate the component concentrations, odor models have been built by regressions. Based on these odor models, a weighted and constrained least-squares problem is solved to estimate the concentration of each of the component present in the examined mixture. A weighting scheme is adopted to reflect the reliability of each estimated sensor response. If the estimated response value of a sensor is close to the observed response, a large weight would be assigned to the squared error between the estimated and observed sensor response.

To evaluate the effectiveness of the proposed methods, a set of odor data has been collected by mixing three highly volatile solvents with different mixing ratios. LDA has been noted for its ability to discriminate between different component sets regardless of its high computational cost. Furthermore, the proposed LWNN method is shown to be comparable to the commonly applied KNN-based methodology but with lower computational cost since there is no additional computation to determine the best K value for better classification performance. However, LWNN is not suitable for estimation of component concentrations and becomes complex when the number of component increases. The proposed methodology that uses a weighted and constrained least-squares method (WCLS) also demonstrates to provide a good estimate for component concentrations especially for odor mixtures, yet WCLS may provide erroneous concentration estimates for pure odors.

## Figures and Tables

**Table 1.** The training stage of LWNN.

**Input:** the training dataset.
**Procedure:**
(1) Start with a set of predefined classes, *Class_1_*, *Class_2_*,…, *Class_N_*. For each class, *Class*_i_,
(2) Compute its centroid $\vec{o_{i}}$ and its associated weighting vector $\vec{w_{i}}$ by Equation (1) and (4).

**Table 2.** The testing stage of LWNN.

**Input:** the testing dataset.
**Procedure:**
For each data point *x⃗*,
(1) Compute the weighted distances between *x⃗* and each of the class centroid $\vec{o_{i}}$ by Equation (2).
(2) Classify *x⃗* as the class whose centroid has the minimum weighted distance to *x⃗*.

**Table 3.** The training stage of WCLS.

**Input:** the training odor dataset.
**Procedure:**
For each pure odor,
(1) Build the pure odor model according to Equation (5).
(2) Build the mixed odor model according to Equation (6).
Compare each of the weighting terms by Equation (9).

**Table 4.** The testing stage of WCLS.

**Input:** the testing odor dataset.
**Procedure:**
For each testing odor data,
(1) Estimate the component concentration by solving a weighted least-squares problem as Equation (8).

**Table 5.** Accuracy of the KNN-based methods.

**Accuracy (%)**	**K = 1**	**K = 2**	**K = 3**	**K = 4**	**K = 5**
KNN	93.94	93.94	*95.45	93.94	93.94
LDA + KNN	96.97	*100.00	98.48	98.48	96.97
PCA + KNN	*48.48	39.39	39.39	40.91	25.76
LWNN	*95.45	—	—	—	—

**Table 6.** The concentration estimation results of the proposed methodology for mixed odors with three components.

	**Real Concentrations**			**Estimated**			
**Component Set**	**M (ppm)**	**E (ppm)**	**A (ppm)**	**M (ppm)**	**E (ppm)**	**A (ppm)**	**RMSE (ppm)**

MEA-1	34	34	34	33.32	30.12	38.61	3.50
MEA-2	34	34	51	37.29	33.34	49.54	2.11
MEA-3	34	34	68	34.69	31.50	68.82	1.57
MEA-4	34	51	34	38.85	49.33	29.74	3.85
MEA-5	34	68	34	35.82	64.28	38.30	3.45
MEA-6	51	34	34	56.11	36.80	26.81	5.34
MEA-7	68	34	34	66.88	29.93	38.24	3.45

**Table 7.** The concentration estimation results of the proposed methodology for mixed odors with two components.

	**Real Concentrations**			**Estimated**			
**Component Set**	**M (ppm)**	**E (ppm)**	**A (ppm)**	**M (ppm)**	**E (ppm)**	**A (ppm)**	**RMSE (ppm)**

ME-1	34	34	0	31.70	31.19	0.46	2.11
ME-2	34	51	0	37.79	45.23	2.84	4.31
ME-3	34	68	0	39.66	64.52	2.67	4.13
ME-4	51	34	0	47.44	32.20	0.00	2.30
ME-5	51	51	0	52.46	47.45	0.00	2.22
ME-6	68	34	0	64.05	31.94	0.00	2.57

EA-1	0	34	34	0.48	32.50	28.64	3.23
EA-2	0	34	51	2.82	28.63	49.35	3.63
EA-3	0	34	68	2.93	24.32	68.49	5.85
EA-4	0	51	34	5.77	39.01	38.21	8.06
EA-5	0	51	51	5.39	37.60	54.68	8.61
EA-6	0	68	34	9.61	48.33	40.86	13.25

AM-1	34	0	34	19.27	13.62	24.29	12.87
AM-2	34	0	51	20.29	7.45	46.22	9.42
AM-3	34	0	68	20.92	2.77	65.17	7.89
AM-4	51	0	34	33.37	7.59	32.14	11.13
AM-5	51	0	51	36.08	3.06	51.03	8.79
AM-6	68	0	34	51.07	1.96	47.15	12.43

**Table 8.** The concentration estimation results of the proposed methodology for pure odors.

	**Real Concentrations**			**Estimated**			
**Component Set**	**M (ppm)**	**E (ppm)**	**A (ppm)**	**M (ppm)**	**E (ppm)**	**A (ppm)**	**RMSE (ppm)**

M-1	28	0	0	7.55	16.20	0.00	15.06
M-2	45	0	0	28.94	11.38	0.00	11.36
M-3	57	0	0	40.94	9.07	0.00	10.65
M-4	68	0	0	53.96	6.57	0.00	8.95
M-5	85	0	0	73.01	3.92	0.00	7.28

E-1	0	28	0	0.02	29.57	0.00	0.91
E-2	0	45	0	1.32	41.71	0.00	2.05
E-3	0	56	0	6.52	48.33	2.49	5.99
E-4	0	68	0	8.00	54.99	4.30	9.16
E-5	0	85	0	6.12	68.56	15.00	13.33

A-1	0	0	28	0.00	20.17	0.00	19.92
A-2	0	0	45	0.00	15.19	18.17	17.80
A-3	0	0	56	0.00	11.64	32.61	15.08
A-4	0	0	68	0.00	7.54	47.22	12.76
A-5	0	0	85	0.00	0.97	71.11	8.04
